# Is differential cleaning needed for SARS-CoV-2 beyond standard procedures? A systematic review

**DOI:** 10.4102/jphia.v16i2.601

**Published:** 2025-01-17

**Authors:** Uduak Okomo, Ememobong N. Aquaisua, Osamagbe Asemota, Deborah Ndukwu, Josephine E. Egbung, Ekpereonne B. Esu, Olabisi A. Oduwole, John E. Ehiri

**Affiliations:** 1 Faculty of Vaccines and Immunity Theme, Medical Research Council Unit, The Gambia at London School of Hygiene and Tropical Medicine, Fajara, The Gambia; 2 Department of Clinical Research, Faculty of Infectious and Tropical Diseases, London School of Hygiene and Tropical Medicine, London, United Kingdom; 3 Department of Health and Demographic Surveillance System, University of Calabar, Calabar, Nigeria; 4 Department of Paediatrics, University of Calabar Teaching Hospital, Calabar, Nigeria; 5 Institute of Tropical Diseases Research and Prevention, University of Calabar Teaching Hospital, Cochrane, Nigeria; 6 Department of Public Health, Faculty of Allied Medical Sciences, College of Medical Sciences, University of Calabar, Calabar, Nigeria; 7 Department of Medical Laboratory Science, Faculty of Medical Laboratory Science, Achievers University, Owo, Nigeria; 8 Mel and Enid Zuckerman College of Public Health, University of Arizona, Tucson, United States

**Keywords:** SARS-CoV-2, environmental cleaning, health facility, infection prevention and control, hospital cleaning, cleaning products

## Abstract

**Background:**

There is a substantial risk of indirect transmission of SARS-CoV-2 from contaminated surfaces and objects in healthcare settings.

**Aim:**

To evaluate the effectiveness of enhanced cleaning protocols for high-touch surfaces during COVID-19, focusing on cleaning products, concentrations, contact time, and recommended frequency.

**Setting:**

We focused on research conducted in healthcare settings or where samples were obtained from healthcare environments.

**Method:**

We assessed studies that compared different cleaning, disinfection, sterilisation, or decontamination procedures and cleaning frequency with standard or routine procedures. We prioritised randomised trials, non-randomised controlled trials, controlled before-and-after studies, and interrupted time series analyses carried out between 01 January 2020 and 31 August 2022.

**Results:**

Three studies met our criteria from 2139 references searched. These studies, which took place in Iran, China and the United States, found that routine terminal cleaning and enhanced terminal cleaning with different cleaning enhancements significantly reduced SARS-CoV-2 surface contamination. One of the studies tested residual SARS-CoV-2 levels after routine and terminal cleaning with varying strengths of disinfectant and evaluated the efficacy of two common types of disinfectants in inactivating SARS-CoV-2 on inanimate surfaces in different hospital wards.

**Conclusion:**

Limited evidence supports cleaning strategies that can reduce the transmission of SARS-CoV-2 from surfaces in healthcare settings. Combining various cleaning methods and using multiple disinfectants can effectively reduce surface contamination.

**Contribution:**

Randomised controlled trials are crucial for evaluating cleaning effectiveness. They must outline cleaning protocols, detailing frequency, product concentration and volume, application methods, soil and surface types, and environmental conditions, to provide strong evidence.

## Background

The Coronavirus disease pandemic of 2019 (COVID-19) was caused by the severe acute respiratory syndrome coronavirus 2 (SARS-CoV-2). By the end of July 2022, SARS-CoV-2 had infected nearly 600 million individuals globally and caused more than 6.4 million deaths.^1^ Available virological and epidemiological evidence shows that SARS-CoV-2 spreads primarily from direct personal contact or exposure to virus-containing airborne respiratory droplets (inhalation or deposition on the mucosae) shed from people infected with COVID-19. The virus is capable of surviving across various surfaces, too, including a wide range of pH values and ambient temperatures, but is susceptible to heat and standard disinfection methods.^2,3^ Despite adherence to current recommendations to limit virus transmission in public and healthcare settings, including physical distancing, the optimum use of face masks and eye protection, basic hand hygiene,^4^ transmission still occurs in the community and healthcare settings.

Transmission of COVID-19 within the healthcare environment represents a severe public health problem.^5^ Contaminated surfaces in the healthcare environment, mainly those touched frequently, act as reservoirs for pathogens and contribute towards pathogen transmission. The healthcare environment is often contaminated when providing care to patients with COVID-19. High concentrations of the SARS-CoV-2 virus have been detected in environmental samples from intensive care units (ICUs) and isolation wards dedicated to caring for patients with COVID-19,^6^ as well as fomites in surfaces, door handles and knobs, used nasal tissues and cell phones in patient rooms.^7^ However, viral ribonucleic acid (RNA) has been inconsistently detected on inanimate surfaces, and the risk of fomite transmission of SARS-CoV-2 is considered low compared with direct contact, droplet transmission or airborne transmission.^8,9^ Procedures that involve contact by a medical device or surgical instrument with a patient’s sterile tissue or mucous membranes also present a major source of infection. Cleaning and disinfection are critical in breaking the cycle of disease and decontaminating surfaces and objects that may have encountered infected patients.^10,11^

Cleaning removes visible soil (e.g. organic and inorganic material) from objects and surfaces and is typically accomplished manually or mechanically using water with detergents or enzymatic products.^11^ Disinfection describes a process that eliminates many or all pathogenic microorganisms, except bacterial spores, on inanimate objects. In healthcare, objects are usually disinfected by liquid chemicals or wet pasteurisation.^11^ Sterilisation is a process that destroys or eliminates all forms of microbial life and is carried out in healthcare facilities by physical or chemical methods such as steam under pressure, dry heat, ethylene oxide (EtO) gas, hydrogen peroxide gas, plasma and liquid chemicals.^11^ Disinfection and sterilisation are essential for ensuring that medical and surgical instruments do not transmit infectious pathogens to patients; however, sterilisation of all patient-care items is not necessary.^11^ Thorough cleaning using appropriate methods is essential before high-level disinfection and sterilisation because inorganic and organic materials on the instruments’ surfaces interfere with these processes’ effectiveness. In the healthcare environment, surfaces are cleaned alone or cleaned and disinfected regularly (e.g. hourly, daily, three times per week) when surfaces are visibly soiled, if there are spillages, and always after patient discharge.^12^ The type and frequency of routine cleaning depend on clinical risk, patient turnover, traffic intensity and surface characteristics.

The evidence for environmental cleaning and disinfection to prevent transmission of pathogens is well established, particularly for methicillin-resistant *Staphylococcus aureus*, vancomycin-resistant enterococcus, *Clostridium difficile* and a range of Gram-negative bacteria.^13^ While the efficacy of different methods to clean and disinfect viruses has been previously studied, there is insufficient evidence regarding whether standard cleaning and disinfection methods in healthcare settings are adequate in the context of COVID-19 infections.^10^ The extent to which these agents work for SARS-CoV-2 in clinical settings is less known. This systematic review aims to summarise the evidence about whether differential cleaning beyond standard environmental cleaning procedures is required for SARS-CoV-2 in healthcare facility settings. The review will also evaluate the optimum frequency of cleaning high-touch surfaces in healthcare settings in the context of COVID-19 and which cleaning products should be used in healthcare settings in the context of COVID-19, including the contact time and concentrations.

## Methods

Because the characteristics of the studies included in this review (study designs, intervention types and outcomes) are too diverse to yield a meaningful summary estimate of the effect, we present the review following the ‘Synthesis Without Meta-analysis’ (SWiM) reporting guideline which is an extension to the Preferred Reporting Items for Systematic Reviews (PRISMA) statement^14^ (see Online Appendix 1 Table S1 – SWiM Checklist).

### Criteria for considering studies for this review

#### Types of studies

We used the Cochrane’s Effective Practice and Organization of Care (EPOC) group guidelines^15^ to consider study include other study designs. We included randomised trials, and where none were available, other designs were considered if they had comparable arms and where at least one arm included a cleaning method other than the named standard practice.

#### Types of settings, interventions and outcomes

This review considered studies conducted in healthcare settings or where samples were drawn from healthcare settings, as this would provide evidence relevant to the review question. We excluded studies evaluating the effects on animal models or *in vitro* conditions, studies conducted in research laboratories, studies simulating healthcare settings and studies on bacterial decontamination rather than viruses, especially SARS-CoV-2. We considered that this approach will allow us to capture studies that are documented in the most widely used databases and will capture studies relevant to the context of the COVID-19 pandemic. For this review, we defined ‘standard’ environmental cleaning procedures (disinfection, sterilisation and decontamination, including cleaning frequency) and cleaning products as ‘all procedures, techniques and products routinely used in any given healthcare setting as outlined in institutional, national, or regional guidelines’. We considered studies that compared a different procedure for cleaning, disinfection, sterilisation or decontamination, and cleaning frequency with the standard or routine procedures. We included a list of the type of cleaning product used and the frequency of cleaning, but this was not adjusted for in the analysis. We excluded descriptive studies on surface contamination that did not involve an assessment of a cleaning intervention (procedure or product).

We considered the following outcomes as relevant: (1) the risk of SARS-CoV-2 infection in humans as defined by the study authors, (2) laboratory-confirmed SARS-CoV-2 on surfaces and materials, (3) residual environmental surface contamination following the use of any cleaning product and procedure and (4) any adverse effects from the cleaning method or product.

#### Electronic searches

We searched the following databases: The Cochrane Library – Central Register of Controlled Trials (CENTRAL) and Cochrane Database of Systematic Reviews; PUBMED, EMBASE, EPOC and Latin American and Caribbean Literature on Health Sciences (LILACS). The searches covered the period from 01 January 2020 to 31 August 2022. We limited our search to studies comparing the effectiveness of routine and differential cleaning procedures and products. No language restriction was applied (see Online Appendix 1 Table S2 – Search strategies). To identify articles that could have been missed in the electronic searches, we checked the reference lists of all included studies and relevant systematic reviews.

#### Selection of studies

The results of the literature search were incorporated into EndNote 20 and deduplicated. After de-duplication, two review authors used the inclusion criteria to screen the titles and abstracts independently from the retrieved search output. About 20% of studies excluded at this stage were screened by a third reviewer as quality control, and any discrepancies were resolved by discussion with a senior reviewer. We obtained the full-text reports for all titles that appeared to meet inclusion criteria or required further analysis for a more detailed eligibility screen by two independent reviewers and then decided on their inclusion. Discrepancies were resolved by discussion among the review team. We recorded the reasons for excluding studies at any stage and outlined the study selection process in a PRISMA diagram.

#### Extraction and management of data

Using standardised forms, two reviewers independently extracted data from each included study. We collected the following information: study design, setting (location and context of the study, type of health facility, availability of cleaning protocol), details about the intervention and comparison, and the outcomes assessed.

#### Risk of bias assessment

The risk of bias for each study was assessed using the Office of Health Assessment and Translation (OHAT) Risk-of-Bias Rating Tool for Human and Animal Studies,^16^ which evaluates the internal validity of included studies – the assessment of whether the design and conduct of the study compromised the credibility of the link between exposure and outcome. Two reviewers independently assessed the following domains of bias for all reported outcomes: (1) selection bias, performance bias, (2) attrition and/or exclusion bias, (3) detection bias and (4) selective reporting bias. Potential sources of bias were graded as low risk (++), probable low risk (+), probable high risk or not reported (–) and high risk (--). Discrepancies between Scores were compared, and any differences were resolved by discussion. The results are presented in a ‘Risk of bias’ assessment table.

#### Measures of intervention effect and data synthesis

The data were not suitable for meta-analysis. The search results and the selection of studies were presented in flow charts and tables that follow the recommendations of the PRISMA and SWiM statements. For any outcomes where it was impossible to calculate an estimate effect, a narrative synthesis was presented, describing the studies in terms of the direction and size of effects and any available measure of precision. The certainty of evidence (CoE) for all outcomes was assessed using the Grading of Recommendations Assessment Development and Evaluation (GRADE) Working group methodology.^17^ The domains considered were risk of bias, consistency, directness, precision and reporting bias. We determined certainty to be high, moderate, low or very low. For each study’s main comparisons and outcomes, we prepared GRADE Summary of Findings tables.^18^

## Results

### Results of the search

**FIGURE 1:**
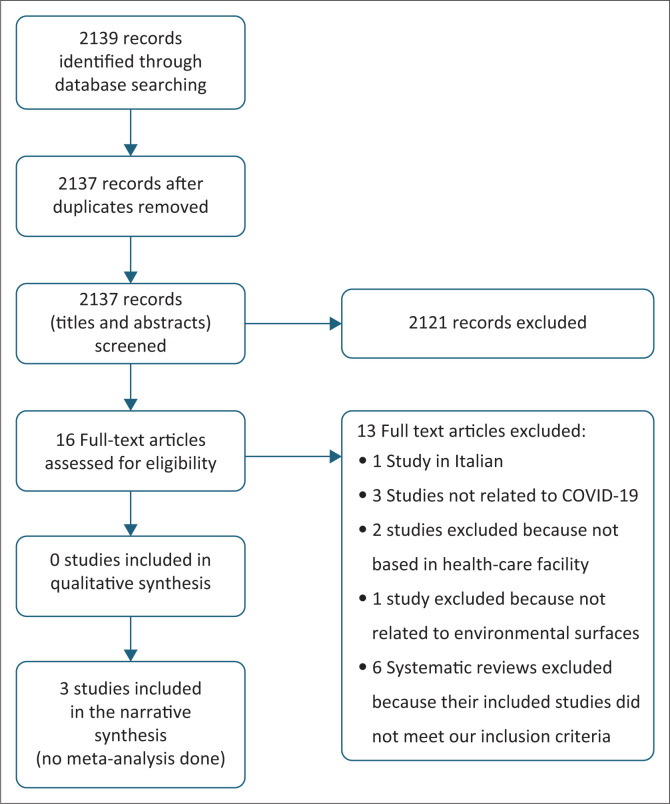
PRISMA flow chart.

The search returned 2139 articles. After de-duplication, we searched the titles and abstracts of 2137 records, of which 16 were eligible for full-text assessment, including six systematic reviews. No randomised trials were identified. We also assessed the full texts of relevant included studies from the six systematic reviews for potentially eligible studies but found none that met our inclusion criteria. Three studies were finally included in the review.^19,20,21^ The details of our search results are presented in a PRISMA flow diagram (Figure 1). The excluded studies are listed in Online Appendix 1 Table S3.

### Description of studies

The included studies were conducted in hospitals in China,^19^ in the United States (US),^20^ and Iran,^21^ in wards and rooms designated for the care of patients with COVID-19. The study settings ranged from single to multi-occupancy rooms where the same surfaces, materials and equipment were sampled before and after cleaning with a disinfectant agent. The study by Ge et al.^19^ was conducted in the isolation wards of a designated COVID-19 Hospital in China. General isolation wards underwent routine cleaning and disinfection three times daily, while isolated ICU wards received these procedures six times daily. Samples were taken 2 h after completing these cleaning activities (Table 1). Routine cleaning and disinfection consisted of wiping 1000 mg/L chlorine-containing disinfectant twice, waiting 30 min, and wiping it with clean water. Visibly contaminated surfaces (including blood and other body fluids) were covered with wipes containing 5000 mg/L chlorine and completely removed before disinfection. High-touch surfaces in examination rooms for COVID-19 patients were sampled after terminal cleaning. The authors reported evaluating the quality of routine disinfection procedures by sampling multiple high-touch surfaces and locations in three areas: the contaminated area, the semi-contaminated area and the clean area. This evaluation was conducted in general isolation and ICU wards before and after cleaning. In their study, Lesho et al.,^20^ evaluated cleaning effectiveness in two different facilities, an Acute Care Hospital (ACH) and a long-term care facility (LTCF) in the US. Nosocomial outbreaks of COVID-19 cases were reported in both facilities. In the ACH, the same eight stationary near-patient, high-touch surfaces were sampled before and after routine terminal cleaning involving surface wiping with a non-bleach sporicidal disinfectant containing hydrogen peroxide and peracetic acid (Table 2). Cleaning effectiveness was also evaluated following the use of three types of terminal cleaning enhancements: Ultraviolet-C (UVC) treatment at 60 000 mJ/cm^2^ (RD™ UVC Mobile System), adding electrostatic spraying (Clorox1Total 3601) following terminal cleaning and adding both UVC and electrostatic spraying. The following enhancements were implemented in addition to terminal cleaning once terminal cleaning was finished. To ensure each enhancement had an equal amount of RNA contamination present before cleaning, 100 cm^2^ surfaces in vacated patient rooms were spotted with 1250 copies of SARS-CoV-2 genomic RNA (BEI Resources) before terminal cleaning. At the LTCF, cleaning effectiveness was evaluated by sampling rooms following routine daily or terminal cleaning by employed cleaning staff and before and after cleaning by a commercial remediation company. In their study, Faezeh and colleagues^21^ examined the efficacy of two common types of disinfectants, alcohol-based hand rubs (ethanol 70%) and sodium hypochlorite (0.001%), to inactivate SARS-CoV-2 on inanimate surfaces in different hospital wards, measured 5 min and 15 min after disinfection (Table 3). The authors did not report details of the cleaning protocols used.

**TABLE 1:** Residual surface contamination with severe acute respiratory syndrome coronavirus 2 following routine disinfection and terminal cleaning protocols in a designated hospital for COVID-19 patients.

Location within hospital	Type of cleaning	Frequency of procedures	Degree of soil	Cleaning agent	Cleaning procedure	Contact time	Proportion of samples with residual contamination
General Isolation wards	Routine disinfection	3x daily	Normal	A total of 1000 mg/L chlorine-containing disinfectant	Clean twice with agent & wait for 30 min then wipe with clean water	30 min	2/105 (%) of samples collected from surfaces in contaminated areas of the hospital.
Isolated ICU wards		6x daily					
General isolation wards	Routine disinfection	3x daily	Visible contaminants including blood and body fluids	5000 mg/L chlorine-containing disinfectant	Cover with disinfectant-soaked wipes and completely remove before disinfection as mentioned above.	30 min	
Isolated ICU wards		6x daily					
Examination rooms	Terminal cleaning	After patient leaves the room	Not stated	Not stated	Not stated	Not stated	0/17

*Source*: Ge T, Lu Y, Zheng S, et al. Evaluation of disinfection procedures in a designated hospital for covid-19. Am J Infect Control. 2021;49(4):447–451. https://doi.org/10.1016/j.ajic.2020.08.028

ICU, intensive care unit.

**TABLE 2:** Residual surface contamination with severe acute respiratory syndrome coronavirus 2 following routine disinfection and terminal cleaning protocols in COVID-19 wards at an Acute Care Hospital and a long-term care facility.

Facility type	Location within the healthcare facility	Type of cleaning	Cleaning agent	Cleaning procedure	Pre-cleaning SARS-CoV-2 surface contamination	Post-cleaning SARS-CoV-2 surface contamination
Acute Care Hospital	COVID-19 Ward	Routine terminal cleaning (employed cleaning staff)	Non-bleach sporicidal disinfectant containing hydrogen peroxide and peracetic acid	Wet mopping and surface wiping (contact time not stated)	67%	22% 65% post-cleaning reduction (*p* = 0.001)
		Enhanced terminal cleaning	Ultraviolet light (UV-C)	Wet mopping and surface wiping followed by UV-C treatment at 60 000 mJ/cm^2^ (RD™ UVC Mobile System) (Contact time not stated)	50%	8.3% 83% post-cleaning reduction (*p* = 0.025)
			Electrostatic spraying (Clorox® Total 360®)	Wet mopping and surface wiping followed by Electrostatic spraying (Clorox® Total 360®) (Contact time not stated)	100%	50% 50% post-cleaning reduction (*p* = 0.006)
			Ultraviolet light (UV-C) + Electrostatic spraying (Clorox® Total 360®)	Wet mopping and surface wiping followed by UV-C treatment at 60 000 mJ/cm^2^ (RD™ UVC Mobile System) and Electrostatic spraying (Clorox® Total 360®) (Contact time not stated)	50%	8.3% 83% post-cleaning reduction (*p* = 0.028)
Long-term care facility	COVID-19 Wards	Routine daily or terminal cleaning (Employed cleaning staff)	None	Light dusting and removal of visible debris	Not stated	Not stated
		Terminal cleaning by commercial remediation company	Three different proprietary room fogging agents (including proprietary chlorine dioxide-based disinfectant)	Room fogging only (contact time in the room ranged from 6 min to 21 min)	Not stated	66%
				Room fogging + terminal-style surface wiping; (contact time in the room ranged from 6 min to 21 min)	19%	17%

*Source*: Lesho E, Newhart D, Reno L, et al. Effectiveness of various cleaning strategies in acute and long-term care facilities during novel corona virus 2019 disease pandemicrelated staff shortages. PLoS One. 2022;17(1):e0261365. https://doi.org/10.1371/journal.pone.0261365

SARS-CoV-2, severe acute respiratory syndrome coronavirus 2; UV-C, Ultraviolet-C.

**TABLE 3:** Persistence of severe acute respiratory syndrome coronavirus 2 on different surfaces after using disinfectants.

Disinfectant type	Inanimate surfaces (location, material and duration of contact)									
	Nursing station (Medium-density fiberboard – MDF)		Reception desk (Wood)		Cell phone (Plastic)		Door handle (Stainless steel)		Floor (Ceramic)	
	5 min	15 min	5 min	15 min	5 min	15 min	5 min	15 min	5 min	15 min
Alcohol-based hand rubs (ethanol 70%)	Positive	Negative	Positive	Positive	Positive	Negative	Positive	Negative	Negative	Positive
Sodium hypochlorite, 0.001%	Positive	Positive	Positive	Positive	Negative	Negative	Negative	Negative	Positive	Positive

*Source*: Faezeh S, Noorimotlagh Z, Mirzaee SA, et al. The SARS-CoV-2 (COVID-19) pandemic in hospital: An insight into environmental surfaces contamination, disinfectants’ efficiency, and estimation of plastic waste production. Environ Res. 2021;202:111809. https://doi.org/10.1016/j.envres.2021.111809

None of the studies described the patients who occupied the rooms during the study period or reported information on the risk of SARS-CoV-2 infection among patients or healthcare workers. Two studies reported the collection and analysis of 122^19^ and 409^20^ and environmental surface samples, respectively. All studies reported on residual surface contamination after cleaning with specific disinfectants described as proportions,^19,20^ or as being simply positive or negative for SARS-CoV-2 viral RNA.^21^ Data were presented for the number of samples collected across the different surfaces. The effectiveness of the employed environmental cleaning methods was reported as a change in viral RNA concentration measured as the proportion of sampled surfaces with detectable SARS-CoV-2 viral RNA after cleaning using threshold Ct values of less than 40 cycles,^21^ and 38 cycles or less.^19^ Although Lesho et al.^20^ assessed the effectiveness of the cleaning by reducing SARS-CoV-2 viral RNA, the threshold Ct value was not reported. All included studies presented crude results without any adjustments in effect measures, and no information was provided to allow for additional analysis in the review. Meta-analysis was, therefore, not possible, and data are presented as narrative summaries.

### Risk of bias in the included studies

None of the studies had serious issues with bias in the selection of surfaces, attrition/exclusion of outcome data, and bias in the selection of the reported results. Table 4 summarises the risk of bias assessment of each domain. The included studies were an experimental study,^21^ single-arm study,^19^ and a controlled before-after study.^20^ Although the studies were all conducted in healthcare settings, it was not possible to pool results in a meta-analysis since there were substantial differences in the location and type of surfaces where samples were collected, interventions and reporting of outcome data. All studies applied a cleaning protocol; however, there was little detail provided to compare these. In one study, an additional cleaning protocol over what is routinely provided was implemented.^20^ Although not directly considered, there were differences in the cleaning practice, frequency and type of disinfectant used across the studies that could influence the outcomes.

**TABLE 4:** Risk of bias assessment.

Study	Study design	Was administered dose or exposure level adequately randomised?	Was allocation to study groups adequately concealed?	Were experimental conditions identical across study groups	Were research personnel blinded to the study group during the study?	Were outcome data complete without attrition or exclusion from analysis?	Can we be confident in the exposure characterisation?	Can we be confident in the outcome assessment (including blinding of assessors)?	Were all measured outcomes reported?	Were there no other potential threats to internal validity?
Ge 2021	Single-arm study	Not applicable	Not applicable	Not applicable	Not applicable	++	++	+	++	+
Lesho 2022	Controlled-before and after study	+	NR	++	Not applicable	++	++	+	++	+
Seif 2021	Experimental study	-	Not applicable	++	Not applicable	++	++	+	++	+

Note: Risk of bias assessment using the Office of Health Assessment and Translation (OHAT) Risk of Bias Rating Tool for Human and Animal Studies Potential source of bias was graded as low risk (++), probable low risk (+), probable high risk or not reported (-).

### Effects of differential cleaning methods, frequencies, cleaning products and contact time

In their study, Ge et al.^19^ reported residual SARS-CoV-2 surface contamination in 1.9% (*n* = 2/105) surfaces routinely cleaned with either a 1000 mg/L chlorine or 5000 mg/L chlorine-containing disinfectant for 30 min, three times or six times daily (Table 1). The certainty of the evidence was rated as very low for routine daily disinfection with 1000 mg/L chlorine compared to routine daily disinfection with 5000 mg/L chlorine (Online Appendix 1 Table S1). No surface contamination was observed following routine terminal cleaning of patient examination rooms; however, cleaning agent, frequency and contact time were not reported.

Lesho et al. reported a 65% post-cleaning reduction (*p* = 0.001) in SARS-CoV-2 surface contamination following routine terminal cleaning (wet mopping and surface wiping) at the ACH.^20^ With enhanced terminal cleaning, the observed post-cleaning reduction in SARS-CoV-2 surface contamination depended on the cleaning enhancement used and ranged from 50% to 83% (Table 2). The certainty of the evidence was rated as very low for routine terminal cleaning compared to enhanced terminal cleaning (Online Appendix 1 Table S2). In the LTCF, where a professional cleaning company was used, SARS-CoV-2 RNA was detected on 66% of surfaces in rooms in which chemical fogging alone was performed but detected on only 17% of surfaces in rooms in which chemical fogging was accompanied by terminal-style surface wiping. The amount of reduction depended on the time spent wiping and the fogging agent used but not the frequency of cleaning. The authors did not report pre- and post-cleaning levels of SARS-CoV-2 surface contamination in wards routinely cleaned daily by employed cleaning staff. However, the certainty of the evidence was rated as very low for routine daily cleaning by employed cleaning staff compared to the professional cleaning company (Online Appendix 1 Table S3).

Fazeh et al.^21^ reported the persistence of SARS-CoV-2 for up to 5 min on most surfaces in the ward and up to 15 min on ceramic floors following disinfection with alcohol-based hand rub (70% ethanol). Severe acute respiratory syndrome coronavirus 2 persisted for up to 15 min on nursing stations, reception desks and ceramic floors following disinfection with 0.001% sodium hypochlorite. The certainty of the evidence was rated as very low for alcohol-based hand rubs (ethanol 70%) compared to 0.001% sodium hypochlorite (Online Appendix 1 Table S4).

## Discussion

We conducted a systematic review and identified three studies that reported data on residual environmental surface contamination with SARS-CoV-2 in different healthcare facility settings and using different cleaning protocols and products. No randomised controlled trials were identified.

Two of the studies,^19,21^ were carried out in hospitals designated for treating patients with confirmed SARS-Cov-2 infection. The third study was a multisite study in an ACH and a LTCF, each with mixed patient populations but with dedicated COVID-19 units for patients with confirmed infection.^20^ Two of the studies field^19,20^ reported on different hospital environmental cleaning protocols (routine daily cleaning and terminal cleaning), while the third assessed the efficiency of two commonly used disinfectants on inanimate surfaces in the hospital.^21^ Routine cleaning methods, environmental sampling methods and SARS-CoV-2 detections varied across studies. The authors of the included studies mention adherence to cleaning protocols by hospital staff or, in one instance, a commercial cleaning company.^20^

One of the studies reported nosocomial outbreaks of COVID-19 cases; however, none directly measured the risk of SARS-CoV-2 transmission from surfaces and materials to humans. The available studies show indirect evidence of the potential risk by sampling surfaces in areas where patients were managed, and these show the presence of viral particles, which can be cleared through various cleaning strategies involving different cleaning agents. Contaminated surfaces can act as a medium for transmitting microorganisms; therefore, disinfection has some benefits in reducing the risk of infection from contaminated surfaces.^22^ These findings emphasise the need to ensure adequate environmental cleaning and establish procedures for correctly disinfection of environments and healthcare equipment that could have been contaminated with SARS-CoV-2.

Healthcare hygiene requires a comprehensive approach, whereby different strategies may be implemented together, in addition to targeted, risk-based approaches, including hand hygiene, cleaning, disinfection, sterilisation and decontamination.^11,23^ Cleaning practices must be tailored to clinical risk, location, site, and hand-touch frequency and evaluated for cost-effectiveness for routine and outbreak situations. In addition to an appropriate spectrum of activity, suitable cleaning/disinfectant products need to comply with occupational health and safety regulations, be approved by regulatory authorities, and be acceptable to users, staff and patients.

This review has several limitations. The main limitation comes from the low quality of the available evidence, as there were no randomised trials. The locations within each healthcare facility, cleaning regimen and environmental surfaces sampled varied widely across the different studies. This makes it challenging to establish the differential efficacy of cleaning protocols and cleaning products in other populations or healthcare settings. The diverse nature of the types of disinfectants and, potentially, cleaning protocols are likely to influence the overall effect of these methods. Environmental surfaces are more likely to be contaminated with SARS-CoV-2 in healthcare settings where specific medical procedures are performed. A systematic review of surface disinfection efficacy studies highlighted variation in experimental conditions as an essential determinant in outcomes.^24^

## Conclusion

Ideally, randomised controlled trials that include a detailed report of the cleaning protocol, frequency of cleaning, volume and concentration of the cleaning product(s) and application mode, soil load, surface type and environmental conditions would be valuable to provide further evidence on this critical topic. Until then, a combination of cleaning methods should be the norm and could yield a more significant reduction in the proportion and volume of infectious contaminants. Using a layered approach to disinfection with multiple disinfectants could contribute to further reductions in surface contamination compared to one method.
